# A framework for the design, conduct and interpretation of randomised controlled trials in the presence of treatment changes

**DOI:** 10.1186/s13063-017-2240-9

**Published:** 2017-10-25

**Authors:** Susanna Dodd, Ian R. White, Paula Williamson

**Affiliations:** 1 Department of Biostatistics, Institute of Translational Medicine, University of Liverpool, Liverpool, L69 3GS UK; 2 MRC Biostatistics Unit, Institute of Public Health, Robinson Way, Cambridge, CB2 0SR UK; 3 MRC Clinical Trials Unit at UCL, Institute of Clinical Trials and Methodology, Aviation House, 125 Kingsway, London, WC2B 6NH UK

**Keywords:** Nonadherence, Non-compliance, Deviation from randomised treatment, Trial analysis, Causal effect modelling

## Abstract

**Background:**

When a randomised trial is subject to deviations from randomised treatment, analysis according to intention-to-treat does not estimate two important quantities: relative treatment efficacy and effectiveness in a setting different from that in the trial. Even in trials of a predominantly pragmatic nature, there may be numerous reasons to consider the extent, and impact on analysis, of such deviations from protocol. Simple methods such as per-protocol or as-treated analyses, which exclude or censor patients on the basis of their adherence, usually introduce selection and confounding biases. However, there exist appropriate causal estimation methods which seek to overcome these inherent biases, but these methods remain relatively unfamiliar and are rarely implemented in trials.

**Methods:**

This paper demonstrates when it may be of interest to look beyond intention-to-treat analysis for answers to alternative causal research questions through illustrative case studies. We seek to guide trialists on how to handle treatment changes in the design, conduct and planning the analysis of a trial; these changes may be planned or unplanned, and may or may not be permitted in the protocol. We highlight issues that must be considered at the trial planning stage relating to: the definition of nonadherence and the causal research question of interest, trial design, data collection, monitoring, statistical analysis and sample size.

**Results and conclusions:**

During trial planning, trialists should define their causal research questions of interest, anticipate the likely extent of treatment changes and use these to inform trial design, including the extent of data collection and data monitoring. A series of concise recommendations is presented to guide trialists when considering undertaking causal analyses.

## Background

### How and why treatment changes occur

Nonadherence with prescribed intervention is a common problem affecting all areas of general medical practice [1, 2]. The wide reaching clinical and economic consequences of nonadherence have prompted extensive research into its causes, extent and impact spanning more than four decades [3]. However, despite these efforts, the prevalence of nonadherence to long-term treatment has remained stable. In a randomised trial, this problem translates into deviation from original randomised treatment, which may be built into, or may contravene, the treatment protocol, depending on the trial’s focus and degree of pragmatism.

Treatment changes are common in trials: a recent review found that 98 of a cohort of 100 trials published in four high-quality general medical journals reported some form of deviation from randomised intervention. However, the analysis methods used to adjust for these changes from randomised treatment were often inadequate [4]. Any deviation from randomised treatment presents a challenge when analysing data. In particular, such deviations impact on the interpretation of trial analyses because the underlying assignment mechanism (randomisation), which forms the basis for unbiased hypothesis testing, no longer reflects the actual treatment received. If all treatment deviations are ignored and analysis is carried out according to randomisation (as in intention-to-treat (ITT) analysis), inference can be made only on the effectiveness of the treatment *policy* or *prescription* in the trial conditions, rather than on the *biological efficacy* (or *causal effect*) of treatment actually received.

### Inherent interest in causal effects

Although ITT is generally recognised as the most appropriate approach for primary analyses of pragmatic clinical trials, mirroring the randomised allocation and thus preventing selection bias, there nevertheless may be interest in estimating efficacy of treatment, or the effectiveness of treatment in an alternative setting to that provided in the trial. The trial may, for practical or ethical reasons, require a certain treatment protocol to be followed; however, the research question of interest may carry an alternative focus to that directly implied by the treatment protocol. For example, crossover to the alternative trial treatment (or non-trial treatments) may be necessary for medical reasons; however, the research question of greatest interest may relate to the effectiveness of treatment in the absence of such treatment switches.

The research question of greatest interest may differ between stakeholders. For example, a motivated patient interested in the likely benefit of treatment if they comply with their prescription may be interested in the causal effect of treatment taken as prescribed while allowing for necessary changes if they experience side effects or treatment inefficacy. Alternatively a policy-maker may be interested in applying trial results to a general community setting where treatment changes occur to differing degrees from those observed in the trial [5]. Differently again, funding decisions by regulatory bodies, such as The National Institute for Health and Clinical Excellence (NICE), often require full cost-effectiveness analysis which typically relate to the effect of treatment taken for life (particularly for drugs which impact on survival). As such, they require estimation of causal effects which factor out all changes from originally randomised treatment that do not reflect typical real-life availability of treatments [6].

### Bias from per-protocol and as-treated analyses

Analysts must be mindful of the potential for bias when analysing according to anything other than randomised allocations, in particular the effect on both internal and external validity of a trial.

Excluding or censoring patients who deviate from randomised treatment protocol (as in ‘per protocol’ (PP) analyses) affects the generalisability (*external* validity) of a trial, as those who persevere with treatment protocol represent a non-random sample of the original group of trial participants. More seriously, PP analysis is likely to introduce selection bias and thus also affect the *internal* validity of a trial. This is because the various treatment protocols being compared present different challenges to adherence, making the compliant subgroups of each randomised group unlikely to be comparable [7]. Given that those intermediate confounding factors which influence a patient’s compliance status as well as their prognosis (and hence outcome) typically remain unmeasured (and may even be unmeasurable), it is often impossible to compare the profiles of these factors between groups. The results of PP analysis are, therefore, likely to be unreliable because of these hidden confounding or selection effects [8].

A variation on PP analysis is analysis according to treatment received (‘as-treated’). Rather than excluding or censoring patients, as-treated (AT) analyses compare patients according to the (predominant) treatment received, but are, therefore, never likely to be valid as randomisation is disregarded entirely [9].

Despite the likelihood of selection bias, these simple methods are frequently used to estimate treatment effects beyond that of ITT; furthermore, analyses purporting to be ‘ITT’ often in fact exclude patients on the basis of treatment adherence, therefore, failing to meet ITT analysis principles [4].

### More appropriate causal methods exist but are rarely used

There exist more appropriate causal estimation methods which seek to overcome the inherent selection and confounding biases of simple methods [10], but these methods remain relatively unfamiliar and are rarely implemented in trials [4]. This may be due to a lack of awareness of how to practically apply the methods, as well as their potential complexity. When planning to implement such methods, it is necessary to consider how the potential treatment deviations will impact on the conclusions drawn from the trial analyses, in relation to the causal research question of interest. Thus, in this paper, we seek to describe a range of trial scenarios where it may be appropriate to look beyond ITT, and to consider the causal research question of interest relative to the treatment deviations observed in the trial. We also discuss issues that must be considered as part of the causal estimation process, in terms of modelling and interpretation of results, and highlight the necessary planning of data collection and statistical analysis methods to ensure transparency and applicability of chosen statistical methods.

### Aim

The aim of this paper is to provide a framework for the design, conduct and interpretation of randomised controlled trials subject to treatment changes, highlighting issues that must be considered at the trial planning stage regarding data collection and analysis beyond ITT. In a complementary paper [11], we demonstrate the application of appropriate causal methods in the analysis of a trial featuring typical treatment complications associated with chronic disease and longitudinal treatment and follow-up periods.

## Framework and recommendations

### Anticipate the possible nature and extent of treatment changes

The first step when planning to carry out causal analysis is to consider how deviations from treatment assignment are likely to manifest themselves, both within the trial setting and the particular (potentially hypothetical) setting to which the trial results are to be applied.

Participant compliance may be all-or-nothing or partial, fluctuating in a time-dependent manner. Treatment switches may take place in one treatment arm only (for example, when control patients are given the option to switch to the experimental treatment on disease progression) or may be very complex (for example, when all patients are permitted to switch to the alternative trial treatment or external non-trial treatments).

In a trial setting, ‘adherence’ comprises more than the patient simply following a prescribed treatment regimen or therapeutic intervention; it also includes cooperation on the part of treatment providers in following the procedures as specified in the treatment protocol. Treatment protocol deviations may also be manifested as (or masked by) absence of outcome data, which is generally caused by withdrawal of patient consent, loss to follow-up (LTFU) or missing assessments. Patient withdrawal from treatment often coincides with premature withdrawal from the trial, as outcome data are often collected at the time of treatment delivery (for example, when patients receive treatment and provide follow-up information at the same clinic visit). Patients may become unavailable for follow-up or withdraw their consent to taking part in the trial for reasons related or unrelated to their condition or treatment.

### Defining causal research question of interest

Once the nature and extent of the likely treatment changes have been identified, it is necessary to define the causal research question of interest, which will in turn allow identification of the causal estimand of interest (that is, the quantity to be estimated). When interest lies in estimating any estimand beyond the effect of treatment assignment, it is important to consider how the treatment patterns in the trial setting relate to this causal estimand. In particular, it is necessary to differentiate those treatment deviations which would be usefully factored out of analysis (because they contravene the treatment path of interest) from those which are inherently part of the treatment course (such that their occurrence is not informative from a causal perspective) [12].

In order to illustrate how treatment changes manifested within a trial scenario may relate to a particular causal research question, we present four varying causal scenarios illustrated using six real-life trials as case studies (see Table 1).

**Table 1:** Case studies illustrating scenarios requiring causal estimation

Causal estimation scenario	Case study	Treatment changes adjusted for	Causal question being addressed
Interest in efficacy despite pragmatic trial design			
	MRC hypertension trial	Addition of alternative treatments due to inadequate blood pressure control, or switches to alternative treatments due to side effects	Treatment efficacy in absence of treatment switches or additions
	SANAD trial	Withdrawal from randomised treatment, addition of alternative treatments, or switches to alternative treatments due to inadequate seizure control	Treatment efficacy in absence of treatment changes due to inadequate seizure control
Trial protocol differs from practice			
	Vitamin A trial	Non-receipt of trial drug due to failure of the drug distribution system	Treatment efficacy among those who would have complied with active treatment if randomised to receive it
Contamination (control arm receives intervention)			
	Honey trial	More extreme forms of treatment, such as antibiotics, surgery or radiotherapy Patient or clinician decision to switch from conventional dressings to honey	Treatment efficacy in absence of any treatment changes
	PACIFICO trial	Switches from standard to new treatment on disease progression	Treatment efficacy in absence of treatment switches from standard to new treatment on progression
Interest in efficacy if taken as prescribed			
	Coronary Drug Project	Failure to take treatment according to prescribed schedule	Treatment efficacy among those who complied with treatment protocol

*MRC* Medical Research Council, *PACIFICO* Purine-Alkylator Combination In Follicular lymphoma Immuno-Chemotherapy for Older patients, *SANAD* Standard and New Antiepileptic Drugs

### Interest in efficacy despite inherently pragmatic trial design

It may be necessary for a trial to be designed with inherently pragmatic characteristics (such as permitting patients to switch from their original randomised treatment if it is found to be inefficacious or unacceptable) in order to mirror usual clinical practice and increase acceptability to participants. However, despite a necessarily pragmatic design, it may be of interest to estimate the underlying efficacy of treatment if taken as originally randomised. In such a case, causal analyses would aim to factor out treatment changes which did not reflect the original intended treatment protocol.

This scenario is particularly likely in the case of trials for chronic conditions, where treatment changes may be common due to problems related to side effects or inefficacy of treatment; two such clinical trial case studies are presented in illustration.

#### MRC hypertension trial

The three-arm Medical Research Council (MRC) hypertension trial [13] compared the long-term efficacy of two antihypertensive drugs (diuretic or beta-blocker) with placebo in prevention of cardiovascular (CV) events and mortality in older patients. The trial protocol permitted changes to randomised treatment, reflecting what would typically occur in clinical practice. If the randomised treatment failed to control blood pressure, an additional drug (initially the other active trial drug) would be prescribed. If a patient experienced unacceptable side effects on randomised treatment, an alternative treatment (commonly the other active trial drug) would instead be prescribed.

The ITT analysis of the MRC hypertension trial appropriately addressed the primary question of interest, namely whether there was any difference in the effectiveness of the treatment policies of starting treatment with one randomised intervention, followed by any treatment changes that became necessary. The effectiveness of the active treatments in preventing CV events compared to placebo was apparent from the ITT analysis; however, when the two active treatment groups were compared directly in an ITT analysis, it was unexpectedly found that the rate of CV events was significantly lower in the diuretic group compared with the beta-blocker group.

Patients randomised to receive a beta-blocker were more often prescribed supplementary drugs than those randomised to diuretic treatment, and they experienced significantly more withdrawals than the diuretic group. In light of the frequency of these treatment changes, the trial investigators explored whether, and how much of, the unexpected ITT difference between the active treatment groups was in fact due to differential rates of treatment changes that occurred in the different randomised groups. White et al. adjusted for changes in prescribed treatment in this trial using both simple [14] and randomisation-based [15] methods (introduced in a complementary paper [11]).

#### SANAD trial

The Standard And New Antiepileptic Drugs (SANAD) trial [16, 17], an unblinded randomised comparison of a number of standard and new antiepileptic drugs (AEDs), is another example of a drug trial in chronic disease which featured changes to treatment prescription, but this trial design was further complicated by the need to balance efficacy and safety. Existing first-line treatments with previously proven efficacy often lead to unacceptable side effects; thus, if a new drug could be shown to be superior in terms of tolerability, it was deemed necessary only to demonstrate its non-inferiority in terms of seizure control (assessed in terms of time to 12-month remission, T12mR, defined as the time from randomisation to reaching a 12-month period free of seizures). The need to employ methods to determine non-inferiority (or equivalence) is complicated when treatment deviations occur, as deviations typically result in merging of treatment experiences across treatment arms, leading to treatment effects that are more similar than would have otherwise been observed between randomised groups. As such, ITT analysis is anticonservative when undertaking such analyses, necessitating estimation methods beyond ITT and PP, as both of these methods are likely to be biased in this setting.

Changes to prescribed treatment are common in epilepsy, primarily due to treatment inefficacy (indicated by inadequate seizure control, ISC) or intolerability (due to unacceptable adverse events, UAEs). Thus, the treatment protocol in SANAD was chosen to be entirely pragmatic, permitting changes from randomised treatment that reflected everyday clinical care. Patients experienced a variety of treatment changes during trial follow-up, including changes to prescribed treatment dose, complete withdrawal from randomised treatment, addition of other trial (or non-trial) treatments to aid seizure control, switching to another trial (or non-trial) treatment or continued prescription of another treatment still being taken at randomisation.

When treatment changes occur prior to achieving remission, the assessment of each randomised AED according to ITT is a distorted assessment of the true efficacy, as it is unclear which AED should be attributed with success or failure in achieving remission. Although it is acknowledged that treatment changes prior to T12mR due to UAEs are inevitable and necessary, expert clinicians argue that changes due to ISC may in fact be avoided by more appropriate dosing of randomised drug. It was, therefore, of particular interest in this trial to supplement ITT analysis with a causal analysis adjusting only for treatment changes occurring due to ISC. The causal question of interest in this trial is: what is the relative benefit of each drug in achieving a minimum T12mR, in the absence of any changes to prescribed treatment due to ISC?

Potential analyses investigating this causal research question are explored in more detail in the complementary paper [11]. Data were available on the nature and timing of changes to prescribed treatment, as well as time-varying covariates that impact on both treatment change and remission, namely seizure counts, adverse events and treatment dose (as the competing reasons for treatment change, ISC and UAE, are inversely related via treatment dose: as dose increases, seizure control is likely to improve but adverse effects may well increase). These data facilitated the use of two different models: the structural failure time model (SFTM, a model based on the potential outcomes framework and the assumed balance between randomised arms in terms of their underlying, potentially unobserved, outcome with the control treatment) and the inverse probability of censoring weighting (IPCW) method (whereby bias introduced with artificial censoring of patients at the point of their first treatment change is addressed by adjusting for all factors that jointly predict treatment change and outcome, under the assumption of no unmeasured confounders).

### Trial protocol differs from what will be used in practice

Another scenario which may necessitate causal analysis is when the treatment protocol implemented in the trial differs from how treatment will be delivered in practice.

#### Vitamin A trial

A cluster randomised trial assessed the effect of treatment with vitamin A (versus no treatment) on infant mortality rates in 450 villages in rural Indonesia [18]. Non-receipt of the trial drug occurred as a direct result of the failure of the trial drug distribution system to reach a substantial proportion of those randomised to receive treatment, rather than due to nonadherence on the part of participants; as such, 20% of the children randomised to receive vitamin A failed to do so.

Investigators were particularly interested in causal analysis beyond ITT because, if proved to be efficacious, the treatment distribution method used in practice to provide vitamin A supplementation to the Indonesian population (fortification of a common daily food) would differ from that used in the trial (oral treatment). Thus, it was considered of greater interest to estimate the biological efficacy of vitamin A supplementation, which could then be interpreted in the light of a likely rate of successful delivery of the chosen vitamin A fortification method, providing an estimate of the effectiveness of the programme to be used in practice, rather than simply the effectiveness of the trial distribution programme (estimated using ITT).

For this trial, Sommer and Zeger [7] proposed a comparison of outcomes among those who would have complied with active treatment if randomised to receive it, later referred to as the complier average causal effect (CACE) estimate. The CACE estimate for the relative risk of infant mortality suggested that vitamin A supplementation would provide more than 70% reduction in infant mortality rates, compared to the ITT relative risk which suggested approximately 40% reduction in risk with orally administered vitamin as distributed in the trial.

### Contamination (whereby control arm receives intervention)

When investigators are interested in comparing the effect of an experimental treatment against a control intervention, the problem of contamination (when participants randomised to control receive the experimental intervention) causes dilution in the estimate of the true efficacy of experimental treatment, as the treatment experience of the two groups becomes more similar than originally intended. In this case, it is of interest to compare the randomised groups factoring out the impact of contamination in the control group. This is a common scenario of interest for health economic evaluations for licensing purposes, as such assessments seek to estimate the cost-effectiveness of treatment compared to the alternative control scenario reflecting a complete lack of availability of the experimental treatment.

Contamination of the control group with the experimental treatment is not uncommon; control participants are made aware of the potential efficacy of the experimental treatment when given information about the trial, and thus it is not surprising that they may seek to obtain the experimental treatment. Furthermore, the trial treatment protocol may permit or encourage treatment switches (for example, on disease progression). Two case studies are presented here, featuring different reasons for contamination in various disease areas.

#### Honey trial

This trial compared the effect of topical medical-grade honey with conventional dressings on wound-healing rates [19]. Despite the trial’s relatively straightforward treatment protocol, a number of deviations from treatment protocol occurred in follow-up.

In cases of deterioration of the wound, it was ethically necessary to allow patients to cease randomised treatment in order to receive more extreme forms of treatment, such as antibiotics, surgery or radiotherapy.

Expectation about honey treatment also led to treatment switches from the randomised conventional arm to honey. Honey was not available on NHS prescription at the time, which (coupled with considerable publicity regarding its efficacy) created a recruitment incentive for both patients and clinical staff. However, the anticipated efficacy of honey, along with the unblinded nature of the trial, frequently led to disappointment and premature dropout when patients were not allocated to receive it.

Treatment switches also occurred because of decisions made by clinical staff external to the trial who were keen for their patients to receive honey, especially their younger, fitter patients expected to make good progress with honey treatment. In contrast, some patients believed that honey treatment was causing additional pain to their wound, which led to a request to switch to conventional treatment.

Although it was necessary to allow patients to receive alternative or more powerful treatment if they experienced side effects or wound deterioration, the primary trial objective was to ascertain the biological efficacy of honey treatment when compared to best standard care. As such, the estimand of interest was the causal effect of honey treatment compared with conventional dressings, factoring out any treatment changes from randomised treatment.

#### PACIFICO trial

Purine-Alkylator Combination In Follicular lymphoma Immuno-Chemotherapy for Older patients (PACIFICO) is a randomised trial (currently recruiting patients) comparing two forms of chemotherapy for patients with follicular lymphoma, assessing which treatment is optimal in terms of efficacy in controlling the spread of the disease balanced against toxicity.

The treatment protocol specifies that cycles of inpatient chemotherapy be delayed or ceased if patients experience excessive toxicity. However, any such deviations from the ideal course of treatment are accepted as an integral part of the variation of treatment according to a patient’s needs and symptoms. In contrast, the treatment changes of interest in terms of causal impact are those made at the point of disease progression. When treating patients for a cancer that may later progress or relapse, it is often an ethical requirement to permit switching to alternative treatments when their disease worsens.

The primary outcome in trials where such treatment changes are expected is usually progression-free survival (PFS) or relapse-free survival (RFS), defined as the time from randomisation to progression (or relapse) or death, whichever occurs first. Such an outcome is not affected by switches at progression (or relapse), as they occur after the event of interest and thus have no causal impact. The traditionally more common outcome of overall survival (OS) is, however, confounded by such treatment changes, as merging of the treatment experience in the two arms following progression or relapse causes diminished OS treatment effects. However, OS is objective and is usually the most important outcome for the patient, as well as being of primary interest for health economics and policy decisions. Thus, on trial completion, it will be of interest to estimate treatment efficacy in terms of OS for this trial using SFTM or IPCW, avoiding confounding due to treatment switches on progression.

### Inform patients and clinicians of efficacy if taken as prescribed

Finally, if trial participants experience problems following the treatment protocol of a treatment which is nevertheless believed or proven to be efficacious, it may be of interest to estimate a causal treatment estimate which factors out the effect of participant nonadherence in order to demonstrate to clinicians and patients the potential optimal efficacy if the treatment prescription is followed as prescribed.

#### Coronary Drug Project

The Coronary Drug Project (CDP) assessed the long-term efficacy and safety of a number of drugs, including clofibrate, against placebo in preventing coronary heart disease (CHD) [20]. Nonadherence to treatment protocol in the CDP occurred as a result of patients failing to take the correct dose of their randomised treatment. Adherence data suggested that one third of the patients (33.5% of the clofibrate group and 32.7% of the placebo group) took less than 80% of their prescribed medication.

Overall clofibrate did not appear to affect mortality when compared to placebo: 5-year mortality in the clofibrate group was 20.0% compared to 20.9% in the placebo group. However, given the high prevalence of non-compliance, the study investigators and external parties became interested in whether the ITT result may have masked a true effect of treatment among those who received clofibrate as intended. Analysis using causal methods, such as CACE estimation to assess the potential effect of treatment among those who ‘complied’ with treatment, would equally be informative for patients wanting to know the benefit of adhering to the relatively complicated treatment schedule.

### Trial designs to address deviation from randomised treatment

As evident in this exposition of trials, deviation from randomised treatment is not always due to a lack of cooperation on the part of the patient or a flaw in the design or methodology of a trial [21]; indeed, nonadherence issues may be inherent to the disease and treatment process. If foreseen, these may be incorporated into the design of a trial during the planning stage. For example, encouragement designs may be employed (whereby encouragement to take the treatment is randomised, rather than the treatment itself) if the consent process is likely to lead to adherence problems. This may be useful if patients are unlikely to accept the idea of randomisation in the given setting or if informing patients about treatment may affect the adherence of patients who end up in the control arm [22].

Other simple design features may be incorporated to aid adherence analyses; for example, compliance measurements may be taken during a baseline placebo run-in phase to obtain information on baseline predictors of compliance (which, when included in CACE and other causal models, may help regain power by reducing outcome variation [23]). Alternatively, it may be possible to seek out patient preferences before they are randomised in order to obtain information on preference effects for better prediction of underlying compliance [24]. Likewise, if a trial is likely to involve numerous forms of treatment change (for example, if a variety of treatment options are available on disease progression) or if extreme degrees of nonadherence are expected (for example, when randomisation is unlikely to be acceptable to most patients), it may be helpful to pre-empt the problem by designing the trial such that specific sequences of treatment are assigned from randomisation, or repeat randomisations take place as and when patients require different treatments. One design of particular importance in chronic conditions where an individual patient’s response may change over time, is the sequential multiple assignment randomised trial (SMART) for the estimation of dynamic treatment regimens [25–27]. SMART designs are particularly appropriate for diseases where sequential phases of treatment are common; for example, chronic conditions like asthma, epilepsy and cancer (requiring different first- and second-line treatments following diagnosis and progression, respectively) and behavioural or psychological interventions. They aim to better estimate the optimal treatment package (sequence of treatments) for *individuals* rather than for *diagnoses*, thus seeking out personalised medicine tailored to suit patients (for example, according to their genetic profile or at a more macro level of characteristics, such as side effect profiles) [28]. However, such designs may themselves be subject to nonadherence (when clinicians prescribe alternatives to the randomised treatment sequences) and may be overly complex or lengthy.

### Appropriate data collection

Having identified which treatment changes impact on the causal estimate of interest, it is necessary to ensure that relevant data on treatment adherence are recorded to facilitate this estimation. Appropriate measurement techniques must be implemented in order to capture information on the particular manifestations of nonadherence that are relevant to the clinical setting, in particular to the disease, treatment and patient population being studied, and the research questions of interest. The measures used to record participant compliance or other treatment changes should be described in sufficient detail to allow assessment of the reliability of the measurements [29].

Adherence or treatment prescription information must be recorded for both treatment arms. Similarly, it may be necessary to supplement data on how well the patients adhere to their original randomised prescription with information on whether they sought alternative treatments or contraindications.

Barriers to accurate collection of treatment adherence data are many, and methods typically used in trials have been discredited, as they are easy to falsify (pill counts), rely on unrealistic or biased recall by patients (patient interviews), or may be resisted by patients (treatment diaries). Similarly, health care providers who (in their opinion, justifiably) deviate from randomised treatment regimens when prescribing or administering patients’ treatment may prefer not to disclose such treatment protocol deviations.

Indeed, trialists may feel overwhelmed when faced with the likelihood of numerous forms of deviation from randomised treatment which typically occur with prescription of long-term medication in trials and in clinical practice. For example, treatment switches, additions, withdrawals (permanent and/or intermittent) and incorrect treatment administration may occur, potentially in both treatment arms and involving trial and non-trial treatments. In such cases, rather than attempting to collect (potentially unreliable) data on all sources of nonadherence to randomised treatment, it will be simpler to identify which features of nonadherence will impact on the outcome, and then focus on how to obtain accurate data on these features alone, which can meaningfully be used to inform relevant analysis techniques. Furthermore, accurate data collection on treatment adherence is often costly, thus adding to the importance of focussed data collection. The complexity and detail of available data will depend on the compliance measurement method. When relying on patient self-report (for example, pill counts, treatment diaries, smartphone apps or direct questioning in clinic), it is crucial that patients are made to feel at ease in reporting their true adherence, rather than being concerned about the consequences of disclosing suboptimal adherence; they must, therefore, be reassured of their valuable contribution when providing accurate adherence information. More objective measures of adherence include medication event monitoring systems (MEMS, which record the exact time and date of opening or activation of drug dispensers [30]), measurement of drug metabolites or markers in bodily fluids and direct observation of therapy (feasible only for monitoring single-dose or intermittent treatment of hospitalised patients rather than long-term self-administered treatment).

The need for relevant adherence data collection highlights the importance of considering causal estimation during the planning stage of a trial, rather than simply at the point of statistical analysis. Given that adherence is typically a multifaceted feature of patient behaviour which is difficult to measure and quantify, it is important to consider which data should be collected and how, such that analysis adjusts for clinically relevant measures of treatment received. The method used to collect compliance data will determine the format of these data and how the data may be included in the model; thus, it will be necessary to consider how to collect this information accurately (considering the potential for measurement errors) and in an unbiased way. In particular, forethought of the likely missingness mechanisms may allow procedures to be employed in order to counter such biases. Furthermore, the complexities associated with recording compliance data mean that it may be useful to pilot any data collection forms prior to trial recruitment in order to ensure sufficient clarity for treatment providers, assessors and patients.

Without the collection of required data, any necessary statistical methods will never be realised; thus, it is important to plan which variables should be collected, relevant to the chosen statistical methods, with consideration of how these compliance and covariate measures will be measured in practice. Statistical analysis may require information, not only on the relevant measures of treatment adherence but also on baseline and post-randomisation time-varying covariates which impact on the decision to change treatment. For example, IPCW methods require adjustment for all known confounders which impact both treatment changes and outcome.

### Reporting and analysis of deviations from treatment protocol

In order to ensure availability of necessary data and protect against selective reporting, trialists need also to consider at the design stage the statistical analysis methods that will be employed to adjust for treatment changes. First, it is necessary to consider the trial aims and complications in interpreting ITT analysis that may be introduced by any anticipated adherence problems in order to determine whether analysis by ITT is likely to be appropriate or sufficient [6]. Regardless of trial aims, however, reporting information on the uptake and acceptance of treatment is important for the interpretation of the success of the trial treatments, even when analysis does not specifically aim to adjust for nonadherence to treatment protocol [4].

### Reporting of compliance data

Even when clinicians are not interested in an explanatory analysis per se (but instead are interested in the effectiveness of the policy of starting with a certain treatment, for example), it is nevertheless important for clinicians to be aware of what changes did occur; otherwise, without an understanding of what the trial treatment policy entailed or how it panned out, it is not possible to fully interpret the effectiveness of the trial treatment policy or to assess the similarity of the trial setting to alternative clinical settings. Even when a trial does not involve many treatment changes, this fact should be communicated so that those interpreting the results are aware that the ITT result is likely to closely mirror the explanatory effect of treatment. Similarly, if a trial is subject to such extreme non-compliance that the results of any analysis are questionable, it is important that the extent of non-compliance is clearly communicated in order that readers appreciate that non-significant results are related to adherence rather than the efficacy of treatment per se. Thus, regardless of whether a trial is designed and analysed to demonstrate effectiveness or efficacy of treatment, or any measure in between, it is important to provide a clear description of the degree and nature of treatment changes.

Adherence information recorded in the trial should be sufficiently detailed and accurate to allow reporting of relevant features of nonadherence which are likely to impact on the course of disease and associated outcomes. Reporting of adherence must also relate to the types of treatment changes expected or encouraged in the trial setting. For example, when treatment switches or additions are likely, it may be relevant to record information not only on patients’ adherence with their original randomised treatment but also with any alternative treatments received, as well as the timing of, and reasons for, such changes [14]. It may also be of relevance to determine who or what was responsible for the decision or request to change treatment prescription (be that the patient, the treating clinician or potentially the protocol itself) and whether this decision was made in a blinded fashion [31].

### Monitoring plan

To this end, it may be helpful to create a monitoring plan which specifies how all relevant compliance data will be collected, recorded and reported during the course of the trial. Data should be collected in order to ensure clinically relevant summaries of treatment adherence can be created [32]. Reporting missing data is as important as disclosure of treatment deviations, given that the two are often related and interlinked [33]. In particular, trial reports should distinguish withdrawal due to LTFU from active decisions to exclude patients from analysis (for example, due to withdrawal or deviation from treatment protocol), which would require appropriate causal methods to avoid subsequent selection bias [34].

### Statistical analysis plan

Consideration of the statistical methods that will be applied to adjust for nonadherence must also take place during the design stage, not only to demonstrate transparency with respect to the planned analyses but also to ensure collection of all necessary information required to facilitate the chosen methods of analysis. This is especially important when considering how to adjust for nonadherence, as adherence is rarely a simple dichotomous measure and may fluctuate within individual over the course of the trial, providing opportunities for manipulation of the particular definition of ‘nonadherence’ in a certain trial in order to produce the most favourable results; for example, by excluding certain patients with particularly good or poor prognoses [35, 36].

As such, in order to avoid accusations of bias, a specific analysis plan should accompany every trial protocol, providing technical details of planned statistical analyses [37]. The choice of statistical methods should be discussed and justified, given that each method has its own different advantages and disadvantages and relies on different assumptions, considering the use of sensitivity analyses to assess departures from identifying assumptions.

Ideally, this plan should include definitions of ‘nonadherence’ and whether, and, if so, how, the efficacy analysis will be adjusted for any nonadherence. These analyses should be linked to the research questions of interest, which then determine the corresponding forms of nonadherence which need to be factored out in order to investigate these questions. This may be a challenging exercise, given the difficulty in predicting all forms of participant or clinician nonadherence that will occur in a trial and, therefore, in defining precisely how particular patients’ data will be analysed (which may explain why, despite the argument for upfront transparency, decisions regarding adherence analyses are often made post hoc) [35]. Indeed Cox [38] argues that although it is necessary to provide a general plan of statistical analysis, it may be unrealistic to require analysts to stick rigidly to specific analysis plans, and that, following analyses carried out according to the original plan, there may be justifiable reasons for making amendments to specific analyses.

Thus, although it may be necessary to make changes to the monitoring or statistical analysis plans during the course of the trial, it is always necessary to disclose the occurrence and reasons for such amendments in order to ensure transparency and accountability.

### Power and sample size

Analysts should consider the effect of nonadherence or treatment changes on the power of trial analyses. Non-compliance in a trial typically reduces the power of ITT analyses because the treatment experiences of randomised groups are more similar than intended. Although it may seem natural to aim to recover this lost power, it is often impossible to do so using the methods discussed above without making additional unverifiable assumptions regarding the comparability of those who do, and do not, comply, such as those underlying PP and AT analyses [5].

For this reason, potential loss of power caused by non-compliance should be considered when planning the sample size of a trial which aims to demonstrate treatment efficacy. Given that it will rarely be possible to regain the associated lost power, the initial sample size should incorporate an inflation factor based on realistic projections of relevant forms of nonadherence or LFTU [39]. If the likely degree of nonadherence is unknown, an adaptive design might be employed, whereby an interim pilot assessment is planned to check the rate of nonadherence midtrial, with the option of increasing the target sample size accordingly [40]. If causal analysis is a secondary objective only, it may be useful to demonstrate the impact of nonadherence on study power for illustrative purposes only.

Snapinn et al. [31] demonstrate how informative non-compliance impacts on sample size and power, discussing different methods to allow for likely non-compliance rates when planning trial sample sizes. The majority of the sample size methods available assume only treatment switches to the alternative treatment and all assume that such discontinuation is independent of outcome (i.e. uninformative). They argue that this (latter) assumption can lead to greatly underestimated sample sizes, because it is not the rate of non-compliance per se, but rather the proportion of *endpoints* occurring in non-compliant patients, that impacts on power; they go on to demonstrate an alternative method of determining sample size which allows for informative dropout [31].

## Discussion

The case studies presented in this paper demonstrate how deviation from randomised treatment may be manifested, in a variety of trial settings, and the subsequent impact on interpretation of analyses. These trials are not unique; the results of a recent review of reporting and analysis of nonadherence in published trials demonstrate clearly that deviation from randomised treatment is a common problem affecting virtually all trials, but that appropriate causal methodology to allow for these deviations is rarely applied [4]. There is, therefore, a pressing need to explore and address the barriers that prevent trial statisticians from applying these more appropriate causal methods to estimate efficacy of treatment when faced with treatment deviations.

The culture of acceptance of ITT as the standard method of analysis, along with a reluctance on the part of trialists to consider alternative methods of analysis out of fear of potentially introducing selection bias, has meant that trials have typically been carried out with a strong focus on pragmatic aims, thus deterring statisticians from investigating or promoting alternatives methods of causal analysis. However, there has recently been an increased awareness (for example, on the part of regulatory or funding bodies) of alternative causal methods which can appropriately address these potential biases [41], and a number of submissions made to NICE [42–46] and the Scottish Medical Consortium [47] have included adjustment for treatment switches.

When such methods are introduced, their sheer complexity, along with a lack of awareness or experience in applying such methods in practice to what may be a complex compliance trial scenario, may hinder their use. In order to implement such methods, it is necessary to recognise how the treatment deviations in a given trial relate to the research question of interest and impact on the conclusions drawn from ITT analysis.

As such, this paper has presented a framework for trialists to follow when considering adjusting for treatment changes in trials, providing examples of when it may be appropriate to consider estimation methods beyond ITT and practical recommendations to ensure such analyses are possible and legitimate. In order that suitable methods of causal analysis can be implemented in practice, it is necessary for researchers to consider certain issues at the design stage of the trial. Appropriate planning is required to ensure the necessary data are collected, not only regarding treatment adherence and outcome, but also on all potential confounding factors that may need to be accounted for. Furthermore, a statistical analysis plan should be developed a priori to ensure that trialists are not accused of altering analysis techniques once outcome data have been collected and observed in order to obtain optimal results.

In a complementary paper [11], we demonstrate how to convert a relatively complex trial compliance scenario into answerable research questions and estimable causal effects. The paper describes the practical application of two causal methods for a complicated time-to-event outcome in the analysis of the SANAD trial, thereby avoiding potential biases associated with commonly used simple methods such as PP or AT analyses.

**Table:** 

Summary of recommendations to trialists: considerations at trial design stage
1. Identify the extent and nature of deviations from randomised treatment that are likely to occur during the course of the trial
2. Specify a priori definition of nonadherence and causal research question of interest, identifying which treatment deviations can be ignored and which should be factored out of analysis
3. Consider whether the expected extent and nature of treatment deviations warrant implementing a particular trial design (such as encouragement design or SMART)
4. Determine necessary data that need to be collected to allow causal analysis to be performed. Data should be collected in order to ensure clinically relevant summaries of compliance can be created, including the reasons for missing data. Collect data on baseline and time-varying confounders (related to occurrence of treatment changes and outcome), including determinants of treatment change. Consider methods to maximise reliability of data (allowing for the potential for distortion or inaccurate recall by patient, or measurement error)
5. Trial reports should clearly communicate the degree and nature of treatment changes, regardless of analysis aims. Create a monitoring plan which specifies how all relevant compliance data will be collected, recorded and reported during the course of the trial. Include in the statistical analysis plan details of proposed statistical analysis methods that will be suitable/possible with the available data to answer the causal research question of interest. However, bear in mind that the analysis plan may need to be amended, subject to complications arising in analysis, in which case causal analyses should be interpreted as exploratory analyses
6. If causal analysis is of primary interest, allow for potential loss of power in sample size calculation, inflating necessary numbers required by a projected percentage of missing outcome data. Use an adaptive design if the likely degree of nonadherence is unknown, with the option to increase target sample size midtrial depending on the rate of nonadherence observed in the interim pilot

## Abbreviations

AT: As treated
CDP: Coronary Drug Project
CV: Cardiovascular
FC: Fludarabine and cyclophosphamide
ISC: Inadequate seizure control
ITT: Intention-to-treat
LTFU: Loss to follow-up
MRC: Medical Research Council
NICE: National Institute for Health and Clinical Excellence
OS: Overall survival
PACIFICO: Purine-Alkylator Combination In Follicular lymphoma Immuno-Chemotherapy for Older patients
PFS: Progression-free survival
PP: Per protocol
RFS: Relapse-free survival
SANAD: Standard and New Antiepileptic Drugs
T12mR: Time to 12-month remission
UAE: Unacceptable adverse effects
